# Supplementary figures and images for: Development of Novel High and Low Emulsifier Diets Based upon Emulsifier Distribution in the Australian Food Supply for Intervention Studies in Crohn’s Disease
Source: Nutrients. 2024 Jun 18;16(12):1922. doi: 10.3390/nu16121922 (PMC11206755; doi:10.3390/nu16121922)

Supplementary Figure 1. Consort diagram for patient flow.

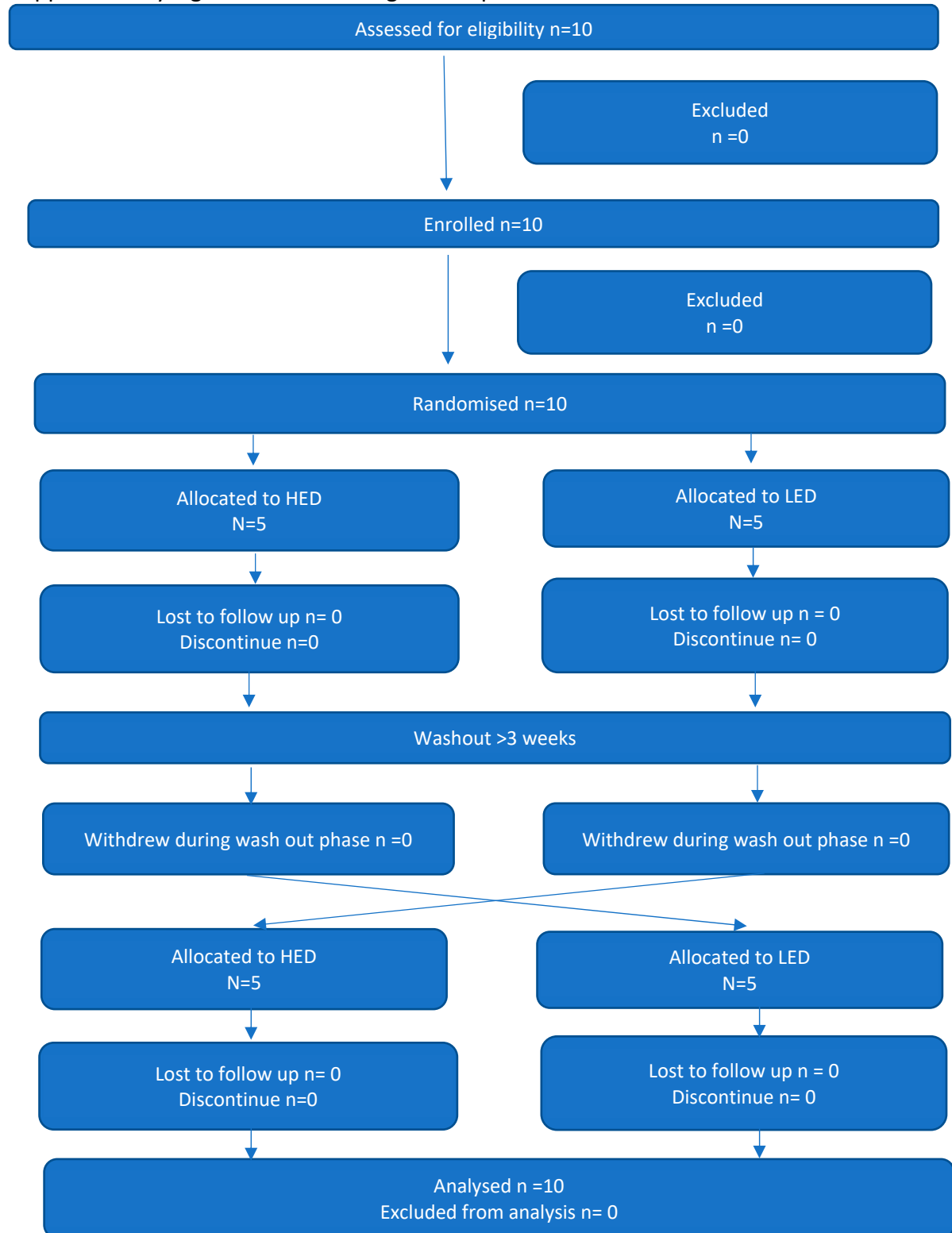

Supplement: Supplementary file 1 [file nutrients-16-01922-s001.zip › Participant flow chart Supplementary.pdf]
